# Malaria epidemiology in Lihir Island, Papua New Guinea

**DOI:** 10.1186/1475-2875-12-98

**Published:** 2013-03-15

**Authors:** Oriol Mitjà, Raymond Paru, Billy Selve, Inoni Betuela, Peter Siba, Elisa De Lazzari, Quique Bassat

**Affiliations:** 1 Department of Medicine, Lihir Medical Center, PO Box 34, Lihir Island, NIP, Papua New Guinea; 2 Barcelona Centre for International Health Research, Hospital Clinic, University of Barcelona, Barcelona, Spain; 3 PNG Institute of Medical Research, Goroka, Papua New Guinea; 4 Sustainable Development Department, Newcrest Mining Ltd, Lihir Island, Put put, Papua New Guinea

**Keywords:** Malaria, Epidemiology, Prevalence, Vector control

## Abstract

**Background:**

*Plasmodium vivax* and *Plasmodium falciparum* malaria remain highly endemic in the Pacific Islands including Lihir Island, Papua New Guinea. Lihir Gold Limited is conducting mining activities and funded an integrated vector control intervention within the villages surrounding the mine. The aim of this study was to assess the impact of such programme by comparing the epidemiological trends of malaria in different parts of the island.

**Methods:**

Two cross-sectional surveys were conducted before and after the intervention (2006–2010) to determine malaria prevalence in mine-impact (MI) and non-MI areas. Incidence of malaria was estimated for the Lihir Medical Centre catchment area using island population denominators and a health-centre passive case detection ongoing from 2006–2011.

**Results:**

A total of 2,264 and 1,653 children < 15 were surveyed in the cross-sectional studies. The prevalence of any malaria parasitaemia initially was 31.5% in MI areas and, 34.9% in non-MI (POR 1.17; 95 CI 0.97 – 1.39). After four years there was a significant reduction in prevalence in the MI areas (5.8%; POR 0.13, 95 CI 0.09–0.20), but reduction was less marked in non-MI areas (26.9%; POR 0.69, 95 CI 0.58-0.81).

28,747 patients were included in the evaluation of incidence trends and overall malaria in local Lihirian population in MI areas declined over time, while it remained at similar high levels among migrants. The age-incidence analysis showed that for each higher age range the malaria incidence declines compared to that of the previous stratum.

**Conclusions:**

There was a substantial reduction in prevalence and incidence rates of both *P. vivax* and *P. falciparum* in the mining area following implementation of a malaria control intervention, which was not seen in the area outside the mining activities.

## Background

The burden of malaria in Papua New Guinea (PNG) is among the highest in Asia and the Pacific region [1,2], and in some areas of the country disease transmission reaches holoendemic levels rarely found outside sub-Saharan Africa. However, the results of a recent country-wide survey, conducted by the PNG Institute of Medical Research (PNGIMR) in 2011, indicate a major reduction in malaria prevalence in all age groups compared to the previous survey [3] as a result of the Global Fund-supported National Malaria Control Programme [4,5]. On average, 6.8% of all surveyed individuals and 7% of all children under the age of five years were infected with malaria parasites as compared to 18% and 24% respectively, in 2008 [6].

The epidemiology of malaria in islands often varies from what occurs in the mainland. Little published data on malaria epidemiology exist for Lihir Island (New Ireland Province), with an extension of 300 square kilometres, and a population of 23,000 inhabitants, and where malaria seems to remain a major public health problem. A general baseline health survey report carried out in 1991 by Bentley *et al.* (unpublised observations) concluded that six of the nine villages surveyed were hyperendemic, and the other three villages were mesoendemic.

Lihir Gold Limited (LGL; Newcrest Mining ltd), a mining company in Papua New Guinea, recognizes that malaria is a major issue affecting employee health as well as the health of communities in areas surrounding their operations in malaria-endemic areas. Therefore, the company is committed to delivering comprehensive and effective management and control of malaria in its sites. Under this premise, the Island’s Public Health Department, planned a malaria control programme specifically targeted at the mine impacted area. This study describes the basic epidemiological data regarding *P. falciparum* and *P. vivax* infections*,* in a coastal area in the northern islands of Papua New Guinea, used for monitoring and evaluating malaria interventions in a mining area.

## Methods

### Study setting and patients

The study was conducted in the main island of the Lihir Group, in the Bismarck Archipelago, 900 km northeast of Port Moresby in the New Ireland Province of Papua New Guinea (PNG). The Lihir islands are geographically remote and host PNG’s largest gold mine since 1995. Mining operations have led to an important increase in the population of the island from 9,900 in 1995 to a population of 21,800 in 2011 of which 6,600 are migrants, and does not include the current mining workforce of 3,500 people (Nick Bainton, Lihir Social impact monitoring programme, personal communication). In the past five years there has been a steep rise in the number of non-Lihirian population, which has increased by 65%. Migrants make their way to Lihir in search of economic opportunities.

The study areas are in a wet tropical forest zone where the mean annual rainfall is about 4,000 mm per annum. There are a large number of streams on the Island, many of which have relatively small catchments with variable flows resulting in fast flowing torrents during heavy rainfall periods and almost no flow during extended dry periods. A drought might cause pooling in rivers and hence create conditions suitable for the breeding of *Anopheles* mosquitoes. The dry season is mainly concentrated between the months of October to December, with the rest of the year being very rainy.

### Vector control intervention

**Figure 1:**
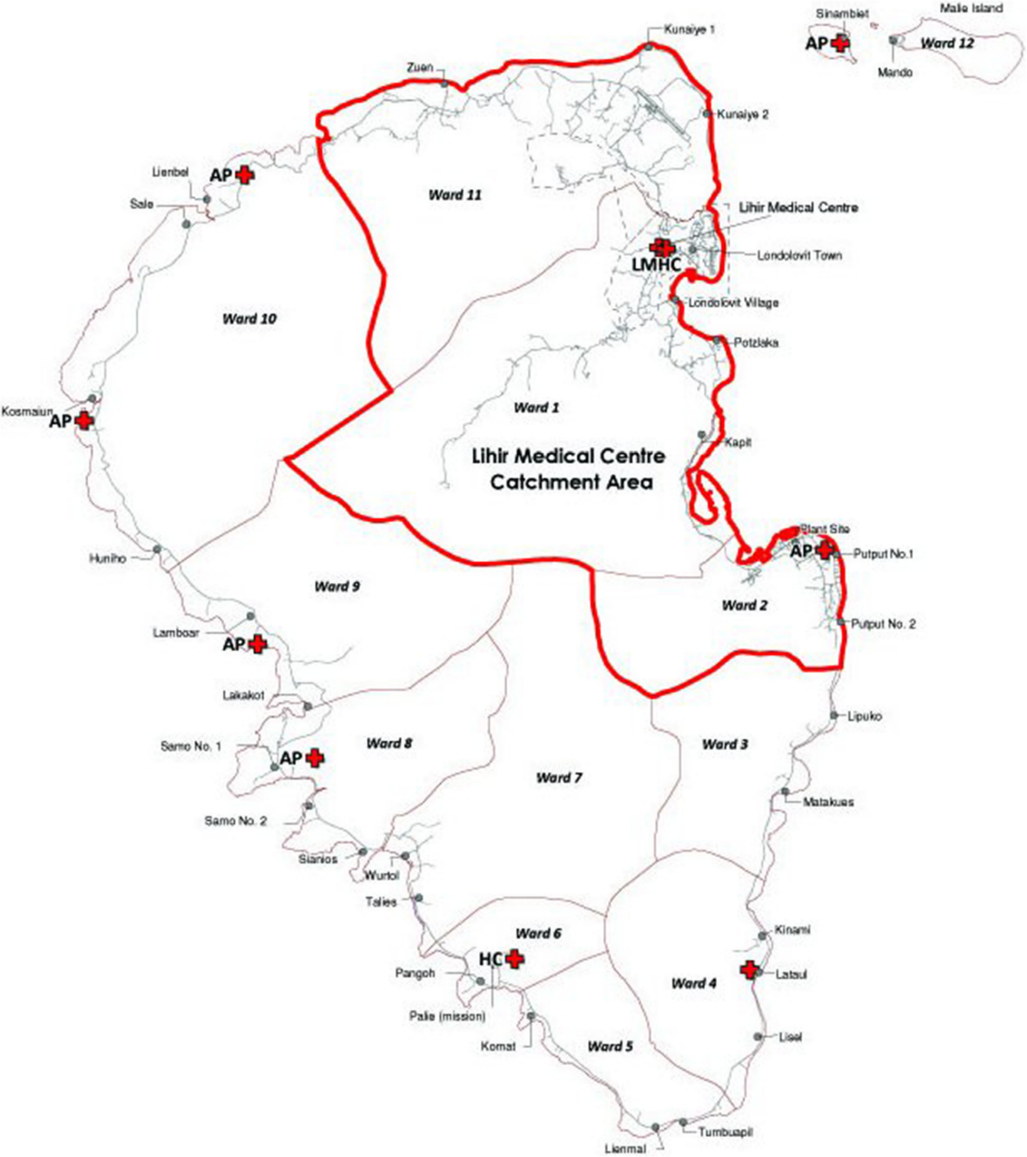
**Map of Lihir Island.** Legend: LMHC = Lihir Medical Health Centre; HC = Health Centre; AP = Aid-Post. Lihir Medical Centre catchment area is the area surrounded by red lines, including all Mine-Impact villages: Zuen, Kunaye 1–2, Londolovit, Kapit, and Put-put 1–2 villages.

The vectors of malaria in New Ireland Province belong to the *Anopheles farauti* and *Anopheles punctulatus* complex of mosquitoes [7,8]. An integrated vector control strategy was put in place in the northern section of Lihir Island in a number of villages between Zuen and Put-put2 affected by mining activities (Figure 1). The programme is based around quantitative monitoring of vectors and interventions include long-term measures targeting adult mosquitoes and mosquito breeding source reduction. Indoor and outdoor residual spraying takes place bi-annually with good coverage of all houses in the mine-impact (MI) area. Distribution of bed nets to all households was implemented in 2004 and again in 2010. Finally, vector breeding site reduction is undertaken once a week around the MI areas; when anophelines are present the area is drained, filled or treated with *Bacillus thuringiensis israelensis.*

### Procedures and data collection

The study involved both active and passive case detection methods for data collection. Two cross-sectional surveys were carried out, in 2006 and in 2010 and the incidence of malaria was measured from January 2006 to December 2011 in the MI area. The annual demographic census of the LGL Social impact monitoring (SIM) report was used to estimate population denominators and incidence rates. Population denominators are well established and updated on continuing basis through quarterly reporting process from village information officers.

#### Active case detection

An island-wide cross-sectional survey with the aim to estimate the community prevalence of malaria parasitaemia in children < 15 years was conducted in January 2006, covering 19 villages as part of a large Mass Drug Administration intervention for filariasis. In January 2010, a second cross-sectional survey in children < 15 years was undertaken covering the same 19 villages. Data were collected by trained field worker teams, which included at least one registered nursing officer for sample collection procedures.

Blood collection was performed by finger-prick. One thick and one thin blood smears were prepared for each survey participant. Thick blood films were examined by light microscopy at Lihir Medical Centre’s microscopy section. Slides were read by WHO-certified microscopists following established standard operating procedures. A slide was recorded as negative when 200 fields of the thick film were read and no parasite was seen. Parasite species in positive films were identified on thin films and gametocyte stages were recorded separately. No molecular techniques were used to complement microscopy. All individuals found to be unwell or parasitaemic during the survey were referred to a study clinician and treated according to PNG National guidelines.

#### Passive case detection

A passive case detection system was used, an administrative database which contains outpatient records of malaria detection at Lihir Medical Centre (LMC). Outpatients visited at LMC from January 2006 through December 2011 were eligible for this study. Patients that resided outside the MI-area (LMC catchment area) (Figure 1) were excluded.

Clinical malaria episodes were defined as a fever (i.e. axillary temperature ≥37.5°C) or history of fever during the preceding 48 hours in the presence of a light microscopically detectable parasitaemia of any *Plasmodium* species and density. Minimum hospital-based incidence rates (MHBIRs) were calculated as the yearly number of malaria cases visited at the outpatient clinic in patients resident in the study area divided by the total individuals’ years at risk (IYAR) for that age group and year. Case-patients were classified as Lihirians or migrants, the latter including all non-Lihirians, both permanent residents and workers living temporarily in and out, in camp accommodation.

All positive malaria cases were treated with appropriate anti-malarial treatment according to the recommendation of the National Malaria Treatment Protocol. Anti-malarial efficacy studies undertaken throughout the 1980s and 1990s in PNG clearly showed the decreasing efficacy of chloroquine (CQ) for the treatment of malaria [9-11]. As a result, the Department of Health protocols for first-line treatment were changed in 1999 to the combination of CQ + sulphadoxine-pyrimethamine (SP), again in 2005 to artesunate (AS) + sulphadoxine-pyrimethamine (SP) and, finally, in 2009 to the combination of artemether + lumefantrine (AL).

#### Entomological surveys

Entomological surveys were carried out in the MI communities from 2006 to 2011. Indoor human landing catches of the vector mosquito were conducted from sunset to sunrise (18:30–06:30 h). With the help of an aspirator, mosquitoes were caught when they landed on a human volunteer for taking a blood meal. This method was approved by local Ethics Committees. The entomologic inoculation rate (EIR) was calculated as the product of the proportion of Anopheles positive by microscopy also termed as sporozoite rate (SR) and the Human Biting Rate, which is estimated as the geometric mean of Anopheles caught per man per month.

#### Rainfall data

The monthly rainfall data for four different meteorological stations from January 2006 to December 2011 were obtained from the LGL Mining department of environment at Lihir. An island-wide rainfall index was constructed based on the records averaged over the four geographically-dispersed stations and diagnostics were reported.

### Statistical analysis

Summary statistics were constructed on the basis of the frequencies and proportions of categorical variables and on the basis of the mean and standard deviation and median and interquartile range (IQR) values of continuous variables. Pearson’s chi-squared test was used to analyse contingency tables, *t*-test for comparing normally distributed quantitative characteristics between groups and, Mann–Whitney *U* test for non-normally distributed quantitative variables. Poisson regression models with robust standard errors were used to estimate the incidence rate ratio (IRR) of malaria outcomes for age ranges and for calendar year. The prevalence odds ratio (POR) for comparing prevalence between groups and between surveys (2010 *vs* 2006) was estimated with a logistic regression model without and with robust standard errors, respectively. Data were entered into Access databases, and Stata version 12.1 (StataCorp) was used for statistical analysis.

### Ethical approval

Ethical approval to conduct the study was granted by the Lihir Sub-district Health Ethics Committee. Community entry involved explaining the study to key community opinion leaders followed by community meetings. At these meetings, the study’s aims, objectives, risk and benefits were explained to all participants.

## Results

### Active case detection

In the 2006 study, a total of 2,264 children were surveyed for malaria parasitaemia. Overall, a total of 760 (33.6% 95 CI 31.7 – 35.5) of all blood slides were found positive. Species distribution included a majority of *P. vivax* (57.0%; 95 CI 53.4 – 60.5) followed by *P. falciparum* infections (40.4%; 95 CI 37.0 – 43.9). *Plasmodium malariae* was less common (2.6%; 95 CI 1.7 – 4.0) and no *Plasmodium ovale* infections were detected in the survey. Malaria parasites where found in 34.9% (95 CI 32.4 – 37.4) of blood slides in the non-MI area and 31.5% (95 CI 28.5 – 34.6) in the MI area (POR 1.17; 95 CI 0.97 – 1.39) (Table 1).

**Table 1:** Prevalence of malaria in children in 19 villages of Lihir Island

	**MI area (%)**	**Non-MI area (%)**	**POR (95% CI)**	**p-value**
2006	31.5	34.9	1.17 (0.97; 1.39)	0.0931
2010	5.8	26.9	5.99 (4.09; 8.77)	<0.0001

**Figure 2:**
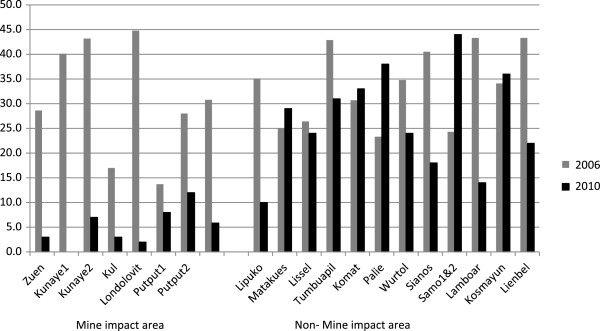
**Malaria prevalence in 19 villages of Lihir Island.** Legend: Results of two cross-sectional surveys in children <15 years. Bars are prevalence of children with malaria parasitemia in every village.

In the 2010 study, a total of 1,653 children from elementary schools were surveyed. Malaria parasites were found in 329 (19.9%, 95 CI 18.1 – 21.9) of all blood slides. Malaria prevalence varied between geographical regions with the highest prevalence found in non-MI villages. (Figure 2) Regional differences for malaria were found to be statistically significant between non-MI and MI areas (26.9 vs. 5.8%, POR 5.99; 95 CI 4.09 – 8.77). Compared to 2006 there was a significant reduction in prevalence in the MI areas (POR 0.13; 95 CI 0.09 – 0.20, P < 0.001) and a less marked reduction in non-MI areas (POR 0.69; CI 0.58 – 0.81, P = 0.012).

### Patient characteristics by passive case detection

From January 2006 to December 2011 a total of 45,594 (mean per annum [SD] 7,599 [1083]) confirmed malaria cases were diagnosed at Lihir Medical Centre (LMC). Of these 45,594 outpatients, 16,847 (37%) patients resided in villages outside the LMC catchment area who either decided to come to the LCM in the first instance, or were referred from a peripheral aid-post or health centre.

The remaining 28,747 patients were included in the study for incidence trends analysis. Table 2 summarizes patient demographic characteristics, clinical severity, and outcomes. Overall, the distribution of male (52.5%, CI 51.9 – 53.0) and female was similar, and the median age of all patients with malaria infection was 20 years (interquartile range [IQR], 5 – 33 years). The median age of patients with *P. vivax* infection was much lower than that of patients with *P. falciparum* (10.0 vs. 22.0 years of age, P < 0.001). A majority of patients were migrants (15,040; 52.3%) as opposed to Lihirians (13,707; 47.7%).

**Table 2:** Characteristics of patients with Malaria infection in the mine-impact area during the study period, 2006 – 2011

**Characteristic**	***P. falciparum *infection (n = 20,302)**	***P. vivax *infection (n = 7,808)**	**All malaria infections (n = 28,747)**
Age, median years (IQR)	22.0 (6.0 – 34.0)	10.0 (3.0 – 30.0)	20.0 (5.0 – 33.0)
Children under 5 years of age, %	18.4% (17.8 – 18.9)	32.6% (31.6 – 33.6)	21.7% (21.3 – 22.2)
Male sex	51.9% (51.2 – 52.6)	53.7% (52.6 – 54.8)	52.5% (51.9 – 53.0)
**Origin**			
Lihirian	46.9% (46.2 – 47.5)	50.3% (49.1 – 51.4)	47.7% (47.1 – 48.3)
Migrant	53.1% (52.5 – 53.8)	49.7% (48.6 – 50.8)	52.3% (51.7 – 52.9)
**Severity and outcome**			
Malaria admissions, n (%)	406 (2.0%; 1.8 – 2.2)	23 (0.3%; 0.2 – 0.5)	429 (1.5%; 1.4 – 1.7)
Died in hospital, n (%)	13 (0.06%; 0.03 – 0.10)	none	13 (0.04%; 0.02 – 0.07)

Legend. Data are % (95 CI) of patients, unless otherwise indicated. *IQR*, interquartile range.

### Temporal trends in malaria infection in Lihirians and migrants

The incidence of malaria infection, *P. falciparum* and *P. vivax* varies significantly over time in all groups (total population, Lihirians, migrants) (Table 3). In the total population and Lihirians, the general trend showed a decline in the incidence over time, but in 2008 compared to 2007 the decline slowed down or converted in an increment, and in 2009 compared to 2008 the incidence was significantly higher. In migrants, *P. falciparum* incidence was almost the same until 2009, in 2010 was significantly higher than 2009 and then in 2011 diminished significantly. *Plasmodium vivax* incidence decreased in 2007 but then increased in 2008 and 2009 and reached a plateau (2010 and 2011).

**Table 3:** Incidence of malaria infection is presented yearly for the total population, Lihirians and migrants

		**2006**	**2007**	**2008**	**2009**	**2010**	**2011**	**p-value**
**All - total population**		n = 7094	n = 7598	n = 8606	n = 10508	n = 10581	n = 10709	
Malaria infection	Incidence	601	494	505	570	542	437	< 0.001
	IRR		0.82	1.02	1.13	0.95	0.81	
**Lihirians**		n = 3594	n = 3660	n = 3768	n = 3905	n = 3980	n = 4059	
Malaria infection	Incidence	723	568	587	740	548	430	< 0.001
	IRR		0.79	1.03	1.26	0.74	0.79	
**Migrants**		n = 3500	n = 3938	n = 4838	n = 6603	n = 6601	n = 6650	
Malaria infection	Incidence	476	425	441	469	538	442	< 0.001
	IRR		0.89	1.04	1.06	1.15	0.82	

Legend. Incidence was only calculated for the mine-impact area. Incidence is estimated as Minimum community-based incidence rates of all microscopically confirmed malaria infections. The Incidence Rate Ratio (IRR) compares the incidences of two consecutive years.

### Malaria species and age trends in burden of disease

Of the total number of malaria parasitologically-confirmed diagnoses in LMC, 70.8% were positive for *P. falciparum*, 27.0% for *P. vivax*, 2.3% for *P. malariae* and 3.2% were diagnosed of mixed infections. *Plasmodium falciparum* asexual stage parasitaemia was much more frequent in sick subjects seen in the health centre than in healthy members of the community when assessed through the cross-sectional surveys in malaria peak season (70.7% vs. 40.4% [OR 3.6; 95 CI 3.26– 3.88, P < 0.001]).

The incidence of malaria infection, *P. falciparum* and *P. vivax* varies significantly over age ranges (Table 4). For each higher age range the incidence declines compared to that of the previous stratum, except for the last one (≥20 years) for which it is higher. For 5–9 years compared to <4 years, the incidence decrease in *P. vivax* is significantly higher than in *P. falciparum,* IRR 0.62 (95% CI 0.58; 0.67) p-value = < 0.0001.

**Table 4:** Incidence of malaria infection stratified for age range

		**≤4 yrs**	**5-9 yrs**	**10-14 yrs**	**15-19 yrs**	**≥20 yrs**	**p-value**
		**n = 538**	**n = 571**	**n = 513**	**n = 402**	**n = 1789**	
**Malaria infection**	**Incidence**	1,264	897	441	317	407	< 0.001
	**IRR (95% CI)**		0.71 (0.47; 1.07)	0.49 (0.47; 0.51)	0.72 (0.67; 0.77)	1.29 (1.21; 1.37)	
***P. falciparum***	**Incidence**	754	622	332	242	308	< 0.001
	**IRR (95% CI)**		0.83 (0.80; 0.85)	0.53 (0.50; 0.56)	0.73 (0.67; 0.79)	1.27 (1.18; 1.37)	
***P. vivax***	**Incidence**	514	262	96	64	88	< 0.001
	**IRR (95% CI)**		0.51 (0.48; 0.55)	0.37 (0.32; 0.41)	0.67 (0.56; 0.81)	1.37 (1.17; 1.62)	

Legend The Incidence Rate Ratio (IRR) compares the incidence of a two consecutive age ranges.

### Seasonality and rainfall

**Figure 3:**
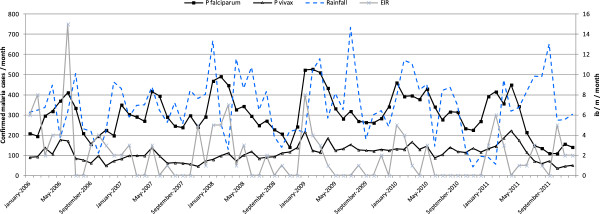
**Monthly confirmed malaria, entomologic inoculation rates and rainfall per month in the period 2006–2011.** Legend: Confirmed malaria cases in the MI area. EIR = entomological inoculation rates in the MI area, calculated as the product of the sporozoite rate (SR) and the Human Biting Rate. Rainfall is an island-wide rainfall index based on the records averaged over 4 geographically-dispersed stations.

Significant seasonality could be observed for *P. falciparum* clinical cases, increasing from January to May and decreasing thereafter. The annual peak of clinical *P. vivax* cases in this period was apparent but less marked. Malaria transmission was highest following the dry season, usually occurring at the end of the year (October - December). Upsurges in malaria transmission were seen with a time lag of one or two months. The average rainfall in the months of October to December was lower (mean [SD] 260.4 [125,8] mm) than the remaining months of the year (mean [SD] 345.7 [149.2] mm per month). The relationship between rainfall and malaria over the time is illustrated in Figure 3.

### Mosquito abundance and man biting rates (MBRs)

A total of 10,061 mosquitoes were captured during the different surveys occurring from 2006 to 2011 in the MI areas, with about a fifth of the mosquitoes belonging to the *Anopheles* species (19.3%; n = 2,323). Non-*Anopheles* species accounted for the remaining 80.7% (n = 9,732), with a predominance of *Culex* and *Aedes* species. EIRs for *Anopheles farauti* were generally low in the MI areas; the median EIR was 1 ib/p/month (IQR 0 – 3 ib/p/month). The highest was recorded in June 2006 and January 2009 (Figure 3).

## Discussion

The study assessed the epidemiology of malaria in a tropical forest area of the New Guinea Islands, PNG, covering villages within a mining area where malaria control interventions have been specifically introduced, and villages outside. Parasite prevalence data was used as the main indicator for transmission reduction. The main findings of this study include the confirmation of a major decrease by 80% in malaria prevalence in the mining area in 2010 compared to the previous survey in 2006. This study has also highlighted the difference in prevalence in villages within the MI areas (6%) compared to those in non-MI areas (27%). There are a number of reasons this may be the case including vector control activities, a better socio-economic profile, better screening or higher use of repellents or easier access to quality care and treatment and to anti-malarial drugs which are more effective in the elimination of the transmissible stage of malaria – gametocytes- than previous drugs used.

When only looking at non-MI areas changes in prevalence of malaria parasitaemia are not substantial. In non-MI villages the prevalence of malaria parasitaemia remained surprisingly high (27%) even when compared to reports of other similar rural parts of the country (6.2% in PNG Islands in 2010) [3]. The reduction in malaria prevalence in other areas of PNG coincided with a major increase in the coverage with long-lasting insecticide treated nets (LLIN) [12]. In Lihir villages, despite the successful distribution of bed nets by the Department of Health in association with Rotarians Against Malaria (36 – 89% of households with at least one bed net) [13], a recent survey by Bentley *et al.* in 2010 showed that the greatest majority of people were not using them for malaria control (unpublised observations).

Incidence (as minimal hospital-based incidence rates) was only calculated for the mining area. The study showed that incidence of malaria in migrant populations, unlike in local populations, has increased in recent years. The poorer living conditions of most migrants (including houses made of bush material, location near swampy areas, and lack of means to buy mosquito nets) put them at continued risk of infection compared to other inhabitants in MI areas [14], but also a migrant infected with malaria can serve as a reservoir becoming an “active transmitter”, thereby enabling the spread of malaria again. The places of origin of migrants are diverse while some are from low transmission sites [15], others are from provinces with historically high rates of malaria [16]. This might indicate that the Island sees a significant arrival of parasites from infected individuals (imported malaria, from highly endemic areas) but also the arrival of individuals with high vulnerability (from low transmission areas) [17,18].

Age-dependent patterns of disease have been observed in this study for *P. vivax* and *P. falciparum* infections. In both cases, the incidence of clinical episodes decreased significantly with age throughout the entire age range until the age of 20 years. However, the peak burden of *P. vivax* infection and illness was concentrated in the younger 0–4 years group, while in *P. falciparum* peak levels remained high until the 10 years of age. Similar results have been reported in other highly endemic areas in PNG where the risk of symptomatic *P. vivax* infection has been found highest among children less than five years of age [19,20]. Under natural, lifelong exposure, immunity to *P. vivax* is acquired more quickly than immunity to *P. falciparum.* With increasing immunity the duration and severity of fevers associated with *P. vivax* infection tend to decrease rapidly, in particular for recurrences with the same strain [19].

There are few longitudinal malaria entomology studies carried out in mining communities in PNG and other parts of the world. Entomologically, only a few malaria vectors can be found on the island of Lihir including *An. punctulatus* found in small numbers relative to *An. farauti sp*., which often breeds in coastal, brackish water [21]. Surveillance conducted along the duration of this study, confirmed the positive impact of vector control on malaria transmission parameters in the MI areas. The EIRs between 2006 – 2010 in the MI area calculated in this survey were relatively low (<1 ib/p/month) as compared to data from the survey by Ebsworth *et al.* in 2001, who estimated EIRs around 10 ib/b/month [21]. The highest EIRs in the MI area were recorded in months that experienced low-moderate rainfall. Low rainfall creates pockets of water, which favours breeding of larvae unlike torrential rains which washes away larval breeding sites of the major malaria vectors in PNG. The association between rainfall patterns and malaria transmission has previously been investigated thoroughly [22,23] and shown once again in this study.

This study has the limitations of a cross-sectional survey; the study captures one moment in time and can hence not consider weather pattern related changes in parasite prevalence. Also, parasite density to determine the expected average decrease as a surrogate marker of the reduction in transmission was not documented. However, both cross-sectional surveys were carried out in the same season. In addition, complementary evidence was provided from incidence measurements and entomological studies suggesting that the results from this survey are indeed a reflection of real and significant changes in the malaria epidemiology. While public-health malaria control oriented activities started by the mining company have resulted in a significant decrease of malaria prevalence in the areas where they have been performed, not much impact has been observed outside these areas. Interestingly, reductions in the malaria burden witnessed by other parts of the country as a result of the scale-up of malaria control interventions have not yet reached Lihir Island, where malaria still represents one of the major public health challenges for the health of its inhabitants.

## Competing interests

The authors declare that they have no competing interests.

## Authors’ contributions

OM conceived the study, supervised data collection and wrote the manuscript. RP and BS led the data collection and participated in data analysis. IB, PS helped to draft the manuscript. EL performed the statistical analysis. QB conceived and designed the study and revised the manuscript. All authors read and approved the final manuscript.
